# The London Poor and Their Medical Needs

**Published:** 1893-04-08

**Authors:** Conrad W. Thies

**Affiliations:** Secretary Royal Free Hospital, London


### THE LONDON POOR AND THEIR MEDICAL NEEDS.

By Mr. Conrad W. Thies, Secretary Royal Free Hospital, London.

(*Concluded from page* 418, *Vol. XIII.*)

After explaining the method by which his inquiries were made, Mr. Booth tells us that "his object was to show the numerical relation which poverty, misery and depravity bear to regular earnings and comparative comfort, and to describe the general conditions under which the people live."

Although I have had rather exceptional opportunities for observing how the poor live, I must confess that, until I had studied Mr. Booth's books, I had not any idea of the extent to which the fell shadow of poverty darkened the lives of such a large proportion of the labouring classes; and I find it difficult, even now, to realise the state of affairs revealed by his statistics, which are, moreover, corroborated by other evidence, to which I can only refer very briefly.

According to the Registrar-General's reports, it appears that about one person in every five of our metropolitan population dies in a pubilc institution, such as hospital, workhouse, infirmary, or asylum. In 1887 the percentage was 20·6 of the total deaths, and in 1888 it rose to 22.3. Considering that comparatively few of these deaths are those of children, it is no exaggeration to say that one person in every four of London adults is driven into one of these public institutions to die, and the proportion in the case of the manual labour classes must, of course, be considerably larger.

The evidence given before the Royal Commission appointed in 1884, "To inquire into the housing of the working classes," revealed a state of things, in the richest city of of the world, which is unworthy of a civilised community. It proved that the single room system for families very largely prevailed, over 200,000 families thus living in one room. In Clerkenwell, very near to where we are now assembled, there are still numbers of tenement houses, each room of which shelters an entire family, sometimes consisting of seven or eight persons. Mr. Booth gives detailed descriptions of many such streets, written by School Board visitors and other persons well acquainted with their respective districts.

There is some difficulty in obtaining full information as to the provision actually existing for the sick poor of the metropolis; but I have summarised the information, which I have obtained from various sources, in the following table:

| | Beds. | In-patients. | Out-patients. |
|---|---|---|---|
| 93 Voluntary Hospitals and Convalescent Institutions | .. 8,032 | .. 82,334 | .. 1,048,536 |
| 9 Hospitals under Metropolitan Asylums Board (for Infectious Diseases | .. 4,122 | .. 16,586 | |
| 24 Poor Law Infirmaries .. | ..12,332 | (no statistics). | |
| Total beds | .. 24,486 | | |

119 Dispensaries, viz.:—

| | |
|---|---|
| 22 Poor Law .. .. .. | (no statistics). |
| 37 Free .. .. .. .. | |
| 16 Part Pay .. .. .. | |
| 44 Provident .. .. .. | |

In the last edition of "Burdett's Hospital Annual" (to which I am also indebted for some of the above figures) there is a very interesting chapter on "The Proportion of In and Out Patients to Population," which is very useful for our present purpose. From the tables given in this chapter it appears that, as regards in-patients London stands eighth on the list, in respect to hospital accommodation, possessing rather less than three beds per thousand, and affording treatment to 25 per thousand of the population; while, in respect of out-patients, it stands second on the list with 417 per thousand receiving treatment. These figures are, however, somewhat misleading, for they are based upon the population comprised in the area of the County of London, viz., 4,221,452, and as I have already pointed out, any calculations in reference to medical relief ought to be based upon the number of the population of Greater London, viz., 5,877,390, and upon this latter basis the metropolis compares very favourably with Birmingham, the proportions of patients to the population being rather less than it is in the Midland town, viz., 18·9 in-patients, and 299 out-patients per thousand respectively. It should, moreover, be borne in mind that the London hospitals provide medical relief to a very considerable number of patients who are sent up for special treatment from the country districts.

Upon comparing the above summary of the numbers of patients treated by the Voluntary Medical Charities, in the light of the information furnished by Mr. Booth, I have come to the conclusion that the accomodation provided is quite sufficient if fully utilised, but is not excessive.

If it is true that about one-third of the entire population of London are comprised in the "poverty classes," that is are subsisting on weekly earnings of less than 21s. for a family, and that often irregularly paid, out of which pittance from 4s. to 7s. must be paid for rent, it is quite evident such people must be ill-nourished and poorly clad, living from hand to mouth, sometimes suffering, sometimes helped by charitable doles; and it needs no argument to prove that they cannot make any provision against "hard times," and are not in a position to pay for medical treatment.

It is most desirable, from the public point of view, that the workers should be afforded every facility for the recovery of health as quickly as possible, and it is impossible for them to receive proper treatment and nursing in their crowded houses.

While these remarks refer mainly to the people comprised in Mr. Booth's "poverty classes," I would point out that the "working classes," who make up rather more than one-half of the entire population, also feel very keenly the pinch caused by sickness; and, indeed, it is from these classes that a very large proportion of the patients of the voluntary hospitals are drawn. Persons earning from 21s. to 50s. per week for a family, while able to pay their way under ordinary circumstances, have not much margin left for making provision against a rainy day, and are little able to bear the extra expenses necessitated by illness, especially when it is the bread winner who is thus laid aside, and the weekly wages are not forthcoming.

The wage limit of 25s. per week for a family has been generally agreed upon in all attempts which have been made to prevent abuse of our medical charities, persons in receipt of less than that sum being considered fit and proper persons for free treatment. Under this wage limit, therefore, all the 1,292,737 comprised in Mr. Booth's "poverty classes" are eligible for free medical relief, and, in addition, a considerable proportion of the 2,166,503 included in the "working classes."

I would ask your attention to another important aspect of this question, which is suggested by the foregoing summary of hospital accommodation in London. I refer to the enormous development which has taken place during the past twenty-five years of the hospitals, infirmaries, and asylums which are supported out of the public funds.

As Mr. Sydney Webb truly says in his "London Programme," "Few people realize how rapidly we are thus 'municipalizing' medical relief." The hospitals and asylums under the control of the Metropolitan Asylums Board and of the Poor Law Authorities have now a total of 16,454 beds. That is more than double the total number of beds provided by all the voluntary hospitals and convalescent institutions put together. These rate-supported institutions for the sick are not exclusively reserved for the use of the poorest classes. In 1887 it was enacted that admission to the public fever hospitals be granted to any person suffering from fever or smallpox whose removal is recommended by a duly qualified medical practitioner. Under this regulation the hospitals of the Metropolitan Asylums Board are yearly becoming more generally used by all classes of the people.

It was stated in evidence given before the Lords' Committee on Poor Law Relief in 1888 that, in consequence of the excellence of the treatment in the Poor Law infirmaries and their separation from the workhouse, the poor are so ready to resort to them in sickness that there is a tendency to regard them as a kind of State hospital, entrance to which does not imply that the patient is a pauper.

It is much to be regretted that the splendid clinical material available in these public institutions is not better utilised in the cause of medical science and education, as most favourable opportunities would thereby be afforded to students for the study of the very classes of disease with which they will so largely have to deal in their future professional work.

I am convinced that this rapid growth of free public hospitals and dispensaries must lead, in the course of the next few years, to some extensive modifications of our present arrangements, and will render it absolutely necessary for the voluntary medical charities to co-operate with each other, if not, indeed, with the rate-supported hospitals. At the present time the voluntary hospitals and convalescent institutions are compelled by their necessities to compete with each other for funds, patients, medical staff, and students.

There is another aspect of this subject, which received prominentt attention by the Lords' Committee in their final report, and to which I will briefly allude, viz., the unequal distribution of the voluntary hospitals. Since the foundation of most of the large general hospitals the population of London has moved further and further away from the central districts, and it has not been found practicable for the hospitals to follow the population. The consequence is, that at the present time, the greater portion of our voluntary hospitals are distributed over London without the slightest regard for local necessities. For instance, within a mile radius of the Middlesex Hospital there are 8 general and 26 special hospitals, with an aggregate of about 2,050 beds, besides 13 dispensaries, all these being in addition to the provision made for the sick poor under the Poor Law. All the voluntary hospitals, in fact, with but few exceptions, lie within an area of about two miles square, while the thickly-populated outlying districts, such as Camberwell and Brixton, are absolutely without any general hospital accommodation.

Mr. Burdett has kindly lent me a map which he has had prepared, and which forcibly illustrate this fact. From his statistics on the subject I have compiled the following table, which shews the distribution of the metropolitan hospitals, taking Charing Cross as a centre.

| — | | Within one mile Radius. | | Within one and two miles Radius. | | Within two and four miles Radius. | | Outside four miles Radius. | |
|---|---|---|---|---|---|---|---|---|---|
| | | No. | Beds. | No. | Beds | No. | Beds. | No. | Beds. |
| Voluntary Hospitals | General... | 5 | 1,477 | 6 | 2,136 | 7 | 1,575 | 4 | 328 |
| | Special ... | 13 | 829 | 9 | 441 | 11 | 1,117 | 2 | 449 |
| Rate Supported Hospitals | M. A. Board | — | — | — | — | 5 | 1,670 | 4 | 1,661 |
| | Poor Law | 1 | 786 | 4 | 2,153 | 9 | 4,126 | 12 | 6,138 |
| Totals .............. | | 19 | 3,092 | 19 | 4,730 | 32 | 8,488 | 22 | 8,576 |

I have refrained on this occasion from any reference to the burning question of the day in the hospital world. I allude, of course, to the recommendation made by the Lords' Committee on Metropolitan Hospitals in their final report for the establishment of a central board. This important suggestion is still under consideration by an influential committee, and we shall, I anticipate, have ample opportunities for considering it before it becomes a question of practical politics. I may, however, venture to remark that the facts I have referred to have a practical bearing on this question, and demand the careful consideration of all who desire to see our great medical charities maintaining their pre-eminence amongst the various efforts which have been put forth to alleviate the hard lot of our poorer neighbours.

There are many other subsidiary questions arising out of the very complex problem of "The London Poor and their Medical Needs," which, had time permitted, I would have touched upon this evening. I trust that the facts I have laid before you, and my remarks thereon, will prove sufficient to provoke a thorough discussion. I am aware that many members of the Hospitals Association are better qualified than myself to express an opinion on this difficult subject, and my paper will have answered its purpose if it stimulates them to give us the benefit of their practical knowledge and experience.

